# A retrospective study on pathological and clinical characteristics of 52 cases with the colorectal serrated polyp

**DOI:** 10.12669/pjms.35.1.238

**Published:** 2019

**Authors:** Xu Li Hua, Li Qian Jun, Shen Xiao Feng, Da Jing, Zhu Ya Wen

**Affiliations:** 1 Xu Li Hua, Department of Gastroenterology, The Sixth People’s Hospital of Nantong, Nantong, Jiangsu-226011, China; 2 Li Qian Jun, Department of Gastroenterology, Huai’an First People’s Hospital, Nanjing Medical University, Huai’an 223300, Jiangsu 226011, China; 3 Shen Xiao Feng, Department of Gastroenterology, The Sixth People’s Hospital of Nantong, Nantong, Jiangsu-226011, China; 4 Da Jing, Department of Gastroenterology, The Sixth People’s Hospital of Nantong, Nantong, Jiangsu-226011, China; 5 Zhu Ya Wen, Department of Gastroenterology, The Sixth People’s Hospital of Nantong, Nantong, Jiangsu-226011, China

**Keywords:** Clinical and Pathological Features, Colorectal Serrated Polyps, Hyperplastic Polyp, Sessile Serrated Adenoma/Polyp

## Abstract

**Background and Objective:**

Colorectal serrated polyp is considered as histologically heterogeneous lesions with malignant potential. The aim of the study was to evaluate the endoscopic, clinic and pathologic characteristics of colorectal serrated polyps.

**Methods:**

The endoscopic, clinic and pathologic characteristics of 52 cases with colorectal serrated polyps between January 2014 and May 2018 in our hospital were analyzed. retrospectively.

**Results:**

The prevalence of serrated polyps was 0.39% (52/13,346). The proportions of hyperplastic polyp (HP), sessile serrated adenoma/polyp (SSA/P), and traditional serrated adenoma (TSA) of all serrated polyps were 61.5%, 17.3%, and 21.2%, respectively, which showed a lower proportion of TSA and SSA/P and a higher proportion of HP.

**Conclusions:**

The overall detection rate of colorectal serrated polyps was relatively low, and it is necessary to discriminate between SSAPs and HPs during endoscopic examination because of the malignant potential.

## INTRODUCTION

Colorectal cancer (CRC) is a major health concern, it is the third most frequent cancer in the world and the fourth most common cause of cancer deaths.^1^ It is currently believed arising from three different pathways: the adenoma to carcinoma pathway which accounts for about 50%-70% of cancers; through the mutator “Lynch syndrome” route (3%-5%); and more recently the serrated pathway (30%-35%).^2^,^3^ Colorectal serrated polyp(SP) is considered as histologically heterogeneous lesions with malignant potential. Colorectal serrated polyps are histologically characteristic for a serrated or “sawtoothlike” appearance of the crypt epithelium^4^, they are classified by the World Health Organization (WHO) into three distinct subtypes: Hyperplastic polyp (HP), sessile serrated adenoma/polyp (SSA/P) with or without cytological dysplasia, and traditional serrated adenoma (TSA).^5^ Unlike adenomas derived from the “adenoma-carcinoma sequence”, not all serrated lesions are linked to colorectal cancer. SSA/P appears to be the responsible precursor lesion for the development of cancers through the serrated pathway.^6^,^7^ Therefore, the aim of our retrospective study was to evaluate the clinical and pathological features of colorectal serrated polyps. We herein reported our experience with 52 cases of colorectal serrated polyps, and evaluated the detection rate and the features of serrated polyps.

## METHODS

This was a retrospective study. All consecutive patients with suspected colorectal serrated polyps who underwent endoscopic treatment at the Digestive Endoscopy Centers of The Sixth People’s Hospital of Nantong and Huai’an First People’s Hospital, between January 2014 and May 2018, were investigated. Lesions that were removed via “cold biopsy” or “hot biopsy” were excluded, because the biopsy material was unsatisfactory (it only included the mucosal surface) and might be not suitable for a pathological diagnosis of SSAP, HP, or TSA. 8 To eliminate any bias due to the diagnostic pathologists, the pathological sections of doubted colorectal serrated polyps were reassessed and reclassified by two experienced pathologists using the WHO criteria. 9 The demographic and clinicopathological data of all colorectal serrated polyps reevaluated with a final diagnosis were further collected, including patient gender, age, lesion size, polyp number, and location. The proximal polyps is defined as the polyps which locate in the cecum, ascending colon, and transverse colon including the splenic flexure, and the distal polyps refer to the polyps in the descending colon, sigmoid colon, and rectum.

Informed consents for colonoscopy were taken from all the patients before the procedure, and ethical approval was obtained from the Ethics Committee of The Sixth People’s Hospital of Nantong and Huai’an First People’s Hospital. 52 patients were enrolled in the study, and a thorough explanation of the procedure was provided to the patients. There were one case of colon carcinoma, two cases of high grade intraepithelial neoplasia, and six cases of low grade intraepithelial neoplasia. Ages of the 52 patients ranged from 22 to 83 years old, and the mean age was 56.6±12.4. The history of presenting problems was abdominal pain in 12 patients (23.1%), hematochezia in 13 (25.0%), abdominal distention in 4 (7.7%) and diarrhea in 9 (17.3%), while the rest of the subjects were discriminated incidentally with endoscopy without specific clinical symptoms.

### Exclusion criteria were as follows: 9,10

1) <22 years old,
2) Patients with any kinds of polyposis syndromes,
3) Patients with a history of CRC or inflammatory bowel disease,
4) Patients with a history of colonic resection or polypectomy,
5) Patients with any emergent and therapeutic colonoscopy,
6) Patients with inadequate bowel preparation and had incomplete colonoscopy.

### Endoscopic procedure and pathological evaluation

Colonoscopy (Olympus CF-Q260, Olympus Optical Co., Tokyo, Japan) was used for all procedures by three experienced endoscopists. All the serrated lesions of the histopathologic specimen database of our hospitals between January 2014 and May 2018 were reassessed and reclassified into three subtypes: HP, SSA/P, and TSA using the WHO criteria.^9^,^10^ The defining pathological feature of HP is a sawtooth pattern of epithelial infolding in the upper half of the crypt with a lack of cytologic dysplasia.^11^ The typical SSA/P has the “boot”, “L”, or “anchor” shaped crypts over the muscularis mucosae, serration in the lower third of the crypts with and without branching of the crypts, inverted crypts under the muscularis mucosae, and columnar dilation in the lower third.^12^ These crypts may appear dilated and/or branched, particularly in the horizontal plane. TSA has the unique characteristics described as a serrated architecture and at least a focal area with tall columnar cells having elongated nuclei and an abundant eosinophilic cytoplasm.

### Statistical analysis

Data were analyzed using the SPSS version 23.0 (SPSS Inc., Chicago IL, USA). Means and standard deviation were calculated for continuous variables. Continuous data were compared using the unpaired Student *t*-test. Categorical variables were examined using the corrected chi-square or two-sided Fisher exact test. P-values of less than 0.05 were considered statistically significant.

## RESULTS

Thirteen thousand three hundred forty six patients undergoing colonoscopy from January 2014 to May 2018 were included in this study and 2,310 (17.3%) patients were detected with at least one colorectal polyp. After the re-classification after the pathological review using the WHO criteria, 52 cases of serrated lesions were found, accounting for 0.39% (52 /13,346) of colonoscopy in the same period, accounting for 2.2% (52/2,310) of the polyp cases detected in the same period. There were 29 males and 23 females in 52 cases. The ratio of male to female was 1.26: 1, with an average age of 56.6±12.4 years old. General clinical epidemiological characteristics of different types of serrated polyps in 52 patients can be seen in Table-I. The average age of HP, SSA/P, and TSA were 58.7±7.8 years, 62.5±11.5 years, and 54.3±12.4 years, respectively. Serrated polyps appeared more in males and elder patients while there was no significant difference in the subtype distribution in gender and age (*P* > 0.05, Table-I).

**Table-I:** General clinical epidemiological characteristics of different types of serrated polyps

Clinical characteristics (n)	CSP(52)	HP(32)	SSA/P(9)	TSA(11)	X^2^/T test	P-value
Age (years)	56.6±12.4	58.7±7.8	62.5±11.5	54.3±12.4		
<50	16	11	1	4	1.639	0.439
≥50	36	21	8	7
*Gender*						
Male	29	20	4	6	0.993	0.642
Female	23	12	5	5
*Symptom*						
Abdominal pain	12	8	2	2	0.02	0.992
Hematochezia	13	7	2	4	0.03	0.972
Abdominal distention	4	2	1	1	0.07	0.963
Diarrhea	9	5	2	2	0.13	0.736
Discovered by examination	14	10	2	2	0.15	0.708

CSP: Colorectal serrated polyps, HP: Hyperplastic polyp, SSA/P: Sessile serrated adenoma/polyp, TSA: Traditional serrated adenoma, SD: Standard deviation.

The endoscopic manifestations of different types of serrated polyps: size, location, shape are listed in Table-II. The serrated polyps in the present study tended to have a small diameter (<20 mm). Furthermore, the location of different types of serrated polyps showed no significant difference in anatomic location (55.8% in the distal colon): HPs (56.3% in distal colon), SSA/Ps (44.4% in distal colon), and TSAs (63.6% in distal colon), respectively. SSAPs were observed more frequently in the proximal colon (oral site from splenic flex), while HP and TSA lesions were observed more frequently in the distal colon (sigmoid colon and rectum). Regarding their gross appearance, most HP and SSAP lesions were flat, while most TSA lesions were protruding. However, there was no significant difference in the study (*P*>0.05, Table-II).

**Table-II:** Endoscopic manifestations of different types of serrated polyps

Endoscopic features (n)	CSP (52)	HP (32)	SSA/P(9)	TSA (11)	X^2^test	P-value
*Location*						
Distal colon	29	18	4	7	0.75	0.733
Proximal colon	23	14	5	4
*Shape^•^*						
0-IIb type	36	26	7	3	2.61	0.486
0-IIa type	10	6	2	2	0.04	0.970
0-Is type	5	0	0	5	-	-
0-Ip type	1	0	0	1	-	-
*Size(mm)*						
≤5mm	16	11	2	3	0.03	0.972
6-10mm	23	16	3	4	0.58	0.742
11-20mm	11	5	3	3	0.18	0.925
<20mm	2	0	1	1	2.21	0.224

CSP: Colorectal serrated polyp, HP: Hyperplastic polyp, SSA/P: Sessile serrated adenoma/polyp, TSA: Traditional serrated adenoma. Proximal colon includes cecum–transverse colon. Distal colon includes descending sigmoid colon.

• The Paris classification of superficial neoplastic lesions in the digestive tract: 0-Ip, 0-Is, 0-IIa, 0-IIb

In total, 17.3% (9/52) serrated polyps were found with dysplasia, and the low-grade dysplasia 66.7% (6/9) was more commonly found. One TSA and one SSAP with high-grade dysplasia were found in the present study. Only one serrated polyp was found associated with canceration. However, there was no significant difference in the study (*P* >0.05, Table-III).

**Table-III:** The degree of dysplasia of each type of colorectal serrated polyp

Dysplasia(n)	CSP	HP(32)	SSA/P (9)	TSA (11)	Fisher exact test	P-value
No dysplasia	43	32	4	7	0.38	<0.05
Low-grade dysplasia	6	0	3	3	0.66	<0.05
High-grade dysplasia	2	0	1	1	0.41	<0.05
Canceration	1	0	1	0	-	-

CSP: Colorectal serrated polyp, HP: Hyperplastic polyp, SSA/P: Sessile serrated adenoma/polyp, TSA: Traditional serrated adenoma.

## DISCUSSION

It has been reported that SSAPs are precursor lesions of sporadic MSI-high (microsatellite instability) cancer, and highly malignant precursor lesions.^2^,^3^ The gross appearance of HPs is similar to that of SSAP endoscopically, though serrated polyps (HPs, SSAP or TSA) can be distinguished pathologically. Therefore, it is necessary to discriminate between SSAPs and HPs during endoscopic examination. After SP being fully recognized and classified in 2005, colorectal serrated polyps were always described to have a flat or sessile appearance and paler than surrounding mucosa under white light colonoscopy.^13^ This may contribute to a high missing rate even if the polyp detection rate and with draw time consistent with standards recommended worldwide. The endoscopy detection rate of SP also varied significantly among different studies.^1^,^4^,^10^ The prevalence of colorectal serrated polyps in western countries ranged from 1% to 18% (average 13%).^2^,^5^,^14^

The current study suggested a lower overall detection rate of colorectal serrated polyps of 0.78% (52 /6673), Compared with the prevalence of conventional adenomas which sharply increased with age^15^,^16^, the age-specific prevalence of serrated polyps was not found to increase with age in the current study (P>0.05) which was dissimilar with other reports.^15^,^17^ However, it is still controversial that some studies have indicated that increasing age was not materially associated with risk of serrated polyps.^18^~^20^ Therefore, further study is needed to determine whether aging is significantly associated with the development of colorectal serrated polyps. Previous studies have reported that large and proximal serrated polyps were the independent predictors of advanced neoplasia.^21^ To investigate the predictors of dysplasia in colorectal serrated polyps, the current study analyzed multiple factors including age, gender, size, location, and dysplasia. We found that HPs are not associated with dysplasia. It is worth paying attention that the SSA/P distribution in this study is not limited to the right semicolon, and in the left semicolon SSA/P also accounts for 44.4% (4/9), suggesting that we cannot ignore partial SSA/P lesions from the left semicolon, and other studies support this view.^6^,^22^

## CONCLUSION

In conclusion, our data showed that the detection rate of colorectal serrated polyps was relatively low, and distribution pattern of the three subtypes is not the same as those reported in the past. Prospective studies may be necessary to evaluate the accurate prevalence and subtypes distribution of colorectal serrated polyps.

### Authors’ Contributions

***Xu Li Hua:*** Design of the study, endoscopy procedures, collection of data and manuscript editing.

***Li Qian Jun:*** Design of the study, works as the first author.

***Shen Xiao Feng:*** Endoscopy procedures, take the responsibility and account for all aspects of the work in ensuring that questions related to the accuracy or integrity of any part of the work are appropriately investigated and resolved.

***Da Jing:*** Statistical analysis and interpretation of the data.

***Zhu Ya Wen:*** Data collection and manuscript writing.
